# Changes in Gene and Protein Expression of Metalloproteinase-2 and -9 and Their Inhibitors TIMP2 and TIMP3 in Different Parts of Fluoride-Exposed Rat Brain

**DOI:** 10.3390/ijms22010391

**Published:** 2020-12-31

**Authors:** Agnieszka Łukomska, Irena Baranowska-Bosiacka, Karolina Dec, Anna Pilutin, Maciej Tarnowski, Karolina Jakubczyk, Wojciech Żwierełło, Marta Skórka-Majewicz, Dariusz Chlubek, Izabela Gutowska

**Affiliations:** 1 Department of Medical Chemistry, Pomeranian Medical University, Powstańców Wlkp. 72 Av., 70-111 Szczecin, Poland; agnieszka_lukomska@wp.pl (A.Ł.); wojciech.zwierello@gmail.com (W.Ż.); marta_skorka@o2.pl (M.S.-M.); 2 Department of Biochemistry, Pomeranian Medical University, Powstańców Wlkp. 72 Av., 70-111 Szczecin, Poland; ika@pum.edu.pl (I.B.-B.); dchlubek@pum.edu.pl (D.C.); 3 Department of Human Nutrition and Metabolomic, Pomeranian Medical University, Broniewskiego 24 Str., 71-460 Szczecin, Poland; deck@cardiff.ac.uk (K.D.); jakubczyk.kar@gmail.com (K.J.); 4 Department of Histology and Embryology, Pomeranian Medical University, Powstańców Wlkp. 72 Av., 70-111 Szczecin, Poland; anna.pilutin@pum.edu.pl; 5 Department of Physiology, Pomeranian Medical University, Powstańców Wlkp. 72 Av., 70-111 Szczecin, Poland; maciej.tarnowski@pum.edu.pl

**Keywords:** MMP2, MMP9, TIMP2, TIMP3, neurotoxicity of fluorine, brain, fluoride, neuroplasticity

## Abstract

Fluoride (F) exposure decreases brain receptor activity and neurotransmitter production. A recent study has shown that chronic fluoride exposure during childhood can affect cognitive function and decrease intelligence quotient, but the mechanism of this phenomenon is still incomplete. Extracellular matrix (ECM) and its enzymes are one of the key players of neuroplasticity which is essential for cognitive function development. Changes in the structure and the functioning of synapses are caused, among others, by ECM enzymes. These enzymes, especially matrix metalloproteinases (MMPs) and their tissue inhibitors (TIMPs), are involved in both physiological processes, such as learning or memory, and pathological processes like glia scare formation, brain tissue regeneration, brain-blood barrier damage and inflammation. Therefore, in this study, we examined the changes in gene and protein expression of MMP2, MMP9, TIMP2 and TIMP3 in the prefrontal cortex, hippocampus, striatum and cerebellum of rats (Wistar) exposed to relatively low F doses (50 mg/L in drinking water) during the pre- and neonatal period. We found that exposure to F during pre- and postnatal period causes a change in the mRNA and protein level of MMP2, MMP9, TIMP2 and TIMP3 in the prefrontal cortex, striatum, hippocampus and cerebellum. These changes may be associated with many disorders that are observed during F intoxication. MMPs/TIMPs imbalance may contribute to cognitive impairments. Moreover, our results suggest that a chronic inflammatory process and blood-brain barrier (BBB) damage occur in rats’ brains exposed to F.

## 1. Introduction

Fluoride (F) compounds accumulating in the central nervous system (CNS) induce neurotoxic effects. In higher concentrations, they lead to the degeneration of brain structures like the hippocampus, cerebral cortex and cerebellum [1]. F could also be accumulated in neurons and astrocytes and causing morphological alterations of these cells [2]. Chronic exposure to F is particularly dangerous in the prenatal period and early life stages, as fluorides are capable of passing through the blood-brain barrier, especially when it is not yet fully developed [3]. F exhibits the ability to pass through the placenta [4] and may cause an adverse effect on fetal brain development. F may also be detected in the mother’s milk during lactation [5].

Prefrontal cortex, hippocampus, striatum and cerebellum are key brain structures due to their function. The prefrontal cortex is involved in the development of working memory, executive functions like planning of movement and regulation of emotion [6]. The hippocampus is important for memory consolidation—transferring information from short-term memory into long-term memory [7]. The striatum is part of the reward system and is necessary for voluntary motor control [8]. The cerebellum is responsible for motor functions, maintaining balance and control of accurate multi-joint movements; it is strongly activated when learning new activities [9].

Both physiological processes, such as the development of cognitive functions, and pathological processes, such as neurodegenerative changes, are accompanied by modifications of synapse structure and function. One of the key components of those processes is proteolysis of the extracellular matrix (ECM), which constitutes the environment for surrounding neurons and glial cells, at the same time serving as a specific modifier of those cells [10]. ECM enzymes, including matrix metalloproteinases (MMPs), are involved in degrading certain ECM proteins, modulating cellular integrity and neuroplasticity [11,12,13,14,15,16]. MMPs are also involved in regulating such processes as cell differentiation and migration, regulating growth factor activity, angiogenesis and inflammation [17,18] by proteolytic degradation of growth factors and cell adhesion molecules [19]. MMP2 and MMP9, belonging to the gelatinase subgroup, play the most crucial role in synaptic plasticity.

Enzymatic remodeling of synaptic connections involving MMP9 and MMP2 is associated with such mechanisms as late-phase of long-term potentiation (LTP) impairment within hippocampal synapses [20,21], changes in dendritic spine morphology in hippocampal neurons [22,23], regeneration of nerve fibers as a result of digestion of damaged ECM components, as well as axon regeneration and elongation [24,25]. Those enzymes are also implicated in morphological changes and maturation of dendritic spines [26,27].

MMP activity and expression are strictly controlled on several levels. One of such control mechanisms is the activity of tissue inhibitors of matrix metalloproteinases—TIMPs [16], among which TIMP2 is the specific inhibitor of MMP2, while TIMP3 exerts a broad-spectrum inhibitory effect against several subgroups of metalloproteinases (including MMP2 and MMP9) and adamalysins (ADAMs). TIMP2 and TIMP3 are also involved in regulating cellular processes, such as cell proliferation, apoptosis and angiogenesis through different mechanisms, not related to MMP inhibition [28,29,30].

Numerous studies on humans [31,32,33,34,35,36,37] and animals [38,39,40,41,42] have shown a positive correlation of long-term exposure to elevated F levels during brain development with reduced intelligence quotient and cognitive impairment. The role played by MMPs/TIMPs in synaptic plasticity, both during prenatal development and in adulthood, is also well documented. Therefore, in this study, we examined the changes in gene and protein expression of MMP2, MMP9, TIMP2 and TIMP3 in the prefrontal cortex, hippocampus, striatum and cerebellum of rats exposed to relatively low F doses during the pre- and postnatal period.

## 2. Materials and Methods

### 2.1. Animal Model of F Intoxication

This study was approved by the Local Ethical Committee for Experiments on Animals in Szczecin, Poland (No. 13/2013, dated 24 October 2013). All procedures involving animals were performed in accordance with the ethical standards of the institution where the studies were conducted.

Experiments were conducted on Wistar rats of both genders. Initially, the females were kept separate from the males and divided into two groups—the experimental group and the control. Then, the females were put in with the males for mating. Pregnant females from the experimental group were given sodium fluoride (NaF) in drinking water at 50 mg/L ad libitum throughout the duration of the pregnancy and afterward, during feeding the offspring. The pups were weaned on postnatal day 26 (PND26) and housed in separated cages. The animals were given a NaF solution (50 mg/L) until PND90. Females from the control group and their offspring were given tap water to drink ad libitum.

After PND90, rats were euthanized under anesthesia (ketamine 80 mg/kg b.w. and xylazine 10 mg/kg b.w.) via decapitation. Each brain was pulled out of the skull and then the prefrontal cortex, striatum, cerebellum and hippocampus were dissected based on the rat brain atlas references [43]. The brain samples (prefrontal cortex, striatum, cerebellum and hippocampus) were immediately frozen in liquid nitrogen and then stored at 80 °C until analysis.

### 2.2. ELISA Analysis of MMP2, MMP9, TIMP2 and TIMP3 Protein Levels

The samples of brain structures (hippocampus, striatum, prefrontal cortex and cerebellum) were homogenized in liquid nitrogen. To achieve cell lysis and release the epitopes of the proteins under analysis, the samples were subjected to three freeze-thaw cycles. The samples were then centrifuged (3000 g/4 °C/20 min) to obtain the supernatant.

Quantitative measurements of MMP2, MMP-9 and TIMP2, TIMP-3 were carried out using ELISA kits (MMP2—Rat matrix metalloproteinase 2/Gelatinase A, Eiaab, cat. no. E0100r; MMP9—Rat Total MMP9 Immunoassay, R&D System, cat. no. RMP900; TIMP2—Rat tissue inhibitors of metalloproteinase 2 ELISA Kit, MyBioSource, cat. no. MBS720098; Tissue Inhibitors of Metalloproteinase 3, ELISA Kit, MyBioSource, cat. no. MBS2514053). The determinations were made according to the manufacturer-supplied manuals using an ASYS UVM 340 microplate reader.

To standardize the obtained results, the total protein content in the samples was measured by a standard bicinchoninic acid (BCA) assay, using Pierce™ BCA Protein Assay Kit. The spectrometric measurement was carried out using an ASYS UVM 340 microplate reader.

### 2.3. qRT-PCR Analysis of MMP2, MMP9, TIMP2 and TIMP3 mRNA Levels

The mRNA expression levels of MMP9, MMP2, TIMP2 and TIMP3 were determined by two-step qRT-PCR analysis. Total RNA was extracted from tissue homogenates by the spin column method using Total RNeasy Mini Kit (Qiagen, Germany) with preliminary DNA digestion. After digesting the residual DNA (RNase-FreeDNase Set, Qiagen, Germany), purifying and eluting the RNA bound to the membrane, spectrophotometric quantification of the isolated RNA (**NanoDrop 1000**, Thermo Fisher Scientific, Germany) was performed. Total RNA was then reverse-transcribed into cDNA (Maxima First Strand cDNA Synthesis Kit for RT-qPCR, Thermo Fisher Scientific, Germany). Copies of the genes of interest were quantified by real-time PCR using the 7500 Fast system and reagents from the Power SYBR Green PCR Master Mix (Thermo Fisher Scientific, Germany). The analysis was repeated twice for each sample. The relative quantity of the genes of interest was normalized in relation to the endogenous reference gene GAPDH. The following primers were used in the procedure: GAPDH—forward primer: ATGACTCTACCCACGGCAAG, reverse primer: CTGGAAGATGGTGATGGGTT; MMP2—forward primer: ACCTGAACACTTTCTATGGCTG, reverse primer: CTTCCGCATGGTCTCGATG; MMP9—forward primer: GAGATGTGCGTCTTCCCCTTC, reverse primer: AGAATGATCTAAGCCCAGCGC; TIMP2—forward primer: TCAGAGCCAAAGCAGTGAGC, reverse primer: GCCGTGTAGATAAACTCGATGTC; TIMP3—forward primer: CTTCTGCAACTCCGACATCGT, reverse primer: GGGGCATCTTACTGAATCCTC).

### 2.4. Immunohistochemistry

The dissected brains from both experimental and control groups were fixed in Carnoy’s fluid (1 h) and then washed in absolute ethanol-xylene serial solutions. The samples were embedded in paraffin blocks and then cut to 3–5 µm serial sections using a microtome (MICROM HM340E and placed on slides (3-aminopropyl-trietoxy-silane, Thermo Scientific, UK)). Sections were deparaffinized and stained with primary antibody against MMP2 (MMP2 (K-20): sc-8835, Santa Cruz Biotechnology, Inc.), MMP9 (MMP9 (C-20): sc-6840, Santa Cruz Biotechnology, Inc.), TIMP2 (TIMP2 (C-20): sc-6835, Santa Cruz Biotechnology, Inc.) and TIMP3 (TIMP3 (W-18): sc-9906, Santa Cruz Biotechnology, Inc.). To visualize the antigen-antibody complex for rabbit antibodies observed under light microscopy (Axioskop Zeiss, Germany), we used DAKO LSAB+System-HRP (DakoCytomation, UK), based on the reaction catalyzed by avidin-biotin-horseradish peroxidase with diaminobenzidine (DAB) as the chromogen. The reaction products were visualized by reacting the tissue with diaminobenzidine (DAB, Sigma-Aldrich, Poland). Images were made using a light microscope (Axioskop Zeiss, Germany) with an integrated camera.

### 2.5. Quantitation

Immunopositive cells were counted under light microscopy (Leica DM5000B, Germany) under the magnification of 40×. Two sections of each brain region per animal were examined for MMP2, MMP9, TIMP2 and TIMP3 immunoreactivity. The number of immunopositive cells per region was calculated using the average of four fields of view per section, and the results were averaged across all the animals in each group (control: *n* = 6, fluorine: *n* = 6) [44].

### 2.6. Statistical Analysis

Statistical analysis of the observed results was carried out using Statistica StatSoft 13.1. After checking data distribution, which was found to deviate from a normal distribution, non-parametric tests were used for further analysis. The Mann–Whitney U test was employed to examine the differences between the study and the control groups. The statistical significance level was set at *p* ≤ 0.05.

## 3. Results

### 3.1. mRNA, Protein Level and Immunolocalization of MMP2 and MMP9

**Figure 1:**
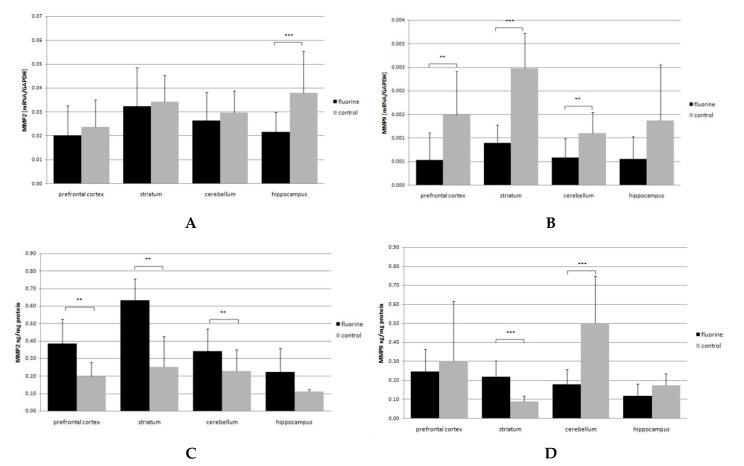
MMP2 and MMP9 mRNA (**A**,**B**) and protein (**C**,**D**) expression in analyzed brain structures (prefrontal cortex, striatum, cerebellum, hippocampus). Black bars represent means ± SD of mRNA (**A**,**B**)/protein (**C**,**D**) level in the Fluoride (F)-exposed group, while gray bars represent controls. ** *p* ≤ 0.005, *** *p* ≤ 0.001 (Mann–Whitney U test), *n* = 6.

MMP2 gene expression in F-exposed animals decreased by 42% in the hippocampus (*p* = 0.00016) (Figure 1A), while the MMP9 gene expression was observed to be lower in the study group than in the control. The most pronounced decrease of the MMP9 mRNA level was found in the prefrontal cortex (67%; *p* = 0.004), striatum (down by 64%; *p* = 0.0002) and cerebellum (down by 45.5%; *p* = 0.009) (Figure 1B).

The level of MMP2 protein was increased in the F-exposed group comparing to the control. For the prefrontal cortex, the enzyme level was 95% higher in the study group (*p* = 0.0002) than in the control. A similar trend was observed in the F-exposed group for the striatum, 152% up (*p* = 0.008), and for the cerebellum, where MMP2 concentration was 48% higher than that in the control (*p* = 0.004) (Figure 1C).

On the other hand, the level of MMP9 protein was lower in the F-exposed group in the cerebellum (*p* = 0.00001) and amounted to as much as 64%. An opposite trend was observed in the striatum, which was the only studied brain structure with the MMP9 level increased by 144.4% (*p* = 0.000003) (Figure 1D).

**Figure 2:**
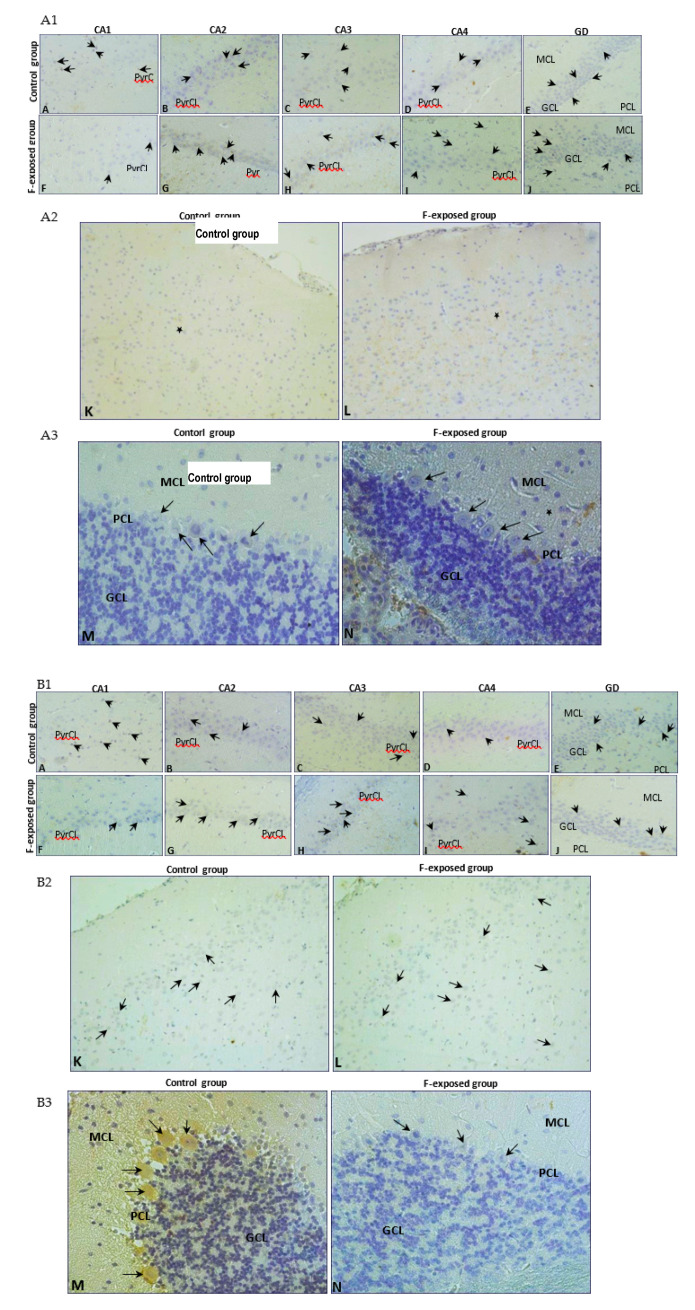
Immunolocalization of MMP2 (**A1**–**A3**) and MMP9 (**B1**–**B3**) in analyzed brain structures (hippocampus, objective magnification ×40 (**A1**,**B1**); prefrontal cortex, objective magnification ×20 (**A2**,**B2**); cerebellum, objective magnification ×20 (**A3**,**B3**)). Hippocampus (CA1–CA4): area of cornu ammonis (CA); gyrus dentate (GD); pyramidal cell layer (PyrCL); granular cell layer (GCL); polymorphic cell layer (PCL); molecular cell layer (MCL).

**Figure 3:**
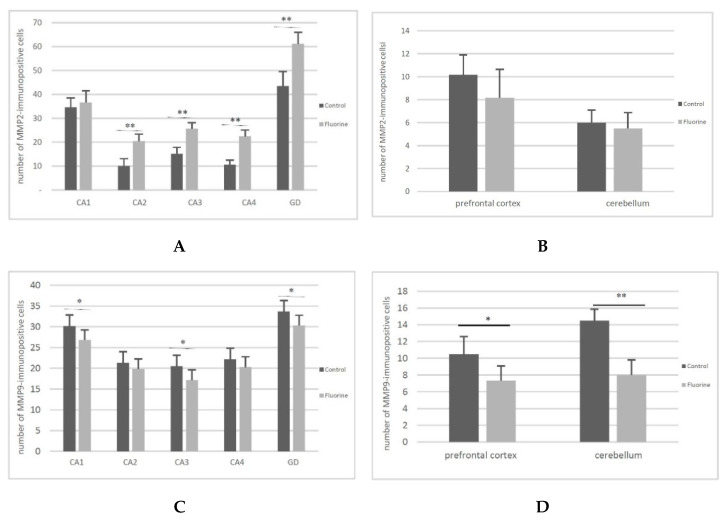
The number of MMP2-immunopositive cells in the hippocampus (**A**) and in the prefrontal cortex and cerebellum (**B**) and the number of MMP9-immunopositive cells in the hippocampus (**C**) and in the prefrontal cortex and cerebellum (**D**). * *p* ≤ 0.05, ** *p* ≤ 0.005 (Mann–Whitney U test), *n* = 6.

Immunohistochemical tests confirmed the findings from the quantitative analysis. All hippocampal regions of both groups showed a positive reaction confirming the presence of MMP2 and MMP9. A stronger MMP2 expression was noted in the CA2-CA4 regions of cornu ammonis (Figure 2, MMP2 G–I, black arrows) and the dentate gyrus (DG) (Figure 2, MMP2 J, black arrows) in the study group compared to the control (Figure 2, MMP2 B–E, black arrows). We also observed a statistically significant increased number of MMP2-immunopositive cells in CA2 (*p* = 0.002), C3 (*p* = 0.002), CA4 (*p* = 0,002) and GD (*p* = 0.002) regions of hippocampus (Figure 3A) in the F-treated group. No significant differences in the MMP9 expression were observed between the control (Figure 2, MMP9 A–E, black arrows) and the study group (Figure 2, MMP9 F–J, black arrows). However, a statistically significant increased number of MMP9-immunopositive cells was observed in CA1 (*p* = 0.026) and CA3 (*p* = 0.041) regions (Figure 3C) in the fluorine group.

In the prefrontal cortex, a stronger MMP2 expression was observed in the cytoplasm of glial cells in the F-exposed group (Figure 2, MMP2 L, black star) compared to the control group (Figure 2, MMP2 K, black star), while there were no significant differences in the MMP2-immunopositive neurons (Figure 3B). MMP9 was expressed in the perinuclear cytoplasm of single neurons in both groups (Figure 2, MMP9 K, black arrows; MMP9 L, black arrows). The number of MMP9-immunopositive cells was significantly decreased (*p* = 0.015) in the F-treated group (Figure 3D).

In the cerebellum, positive staining localized MMP2 was found in the cytoplasm of Purkinje cells of both groups (Figure 2, MMP2 M, black arrows; MMP2 N, black arrows), and there were no statistically significant differences in the number of MMP2-immunopositive cells (Figure 3B).

Strong expression of MMP9 was visible in the cytoplasm of Purkinje cells in the control (Figure 2, MMP9 M, black arrows), while the cytoplasm of Purkinje cells in the experimental group was characterized by markedly lower expression of MMP9 (Figure 2, MMP9 N, black arrow). The number of MMP9-immunopositive cells was significantly decreased (*p* = 0.002) in the fluorine group (Figure 3D).

### 3.2. mRNA, Protein Level and Immunolocalization of TIMP2 and TIMP3

**Figure 4:**
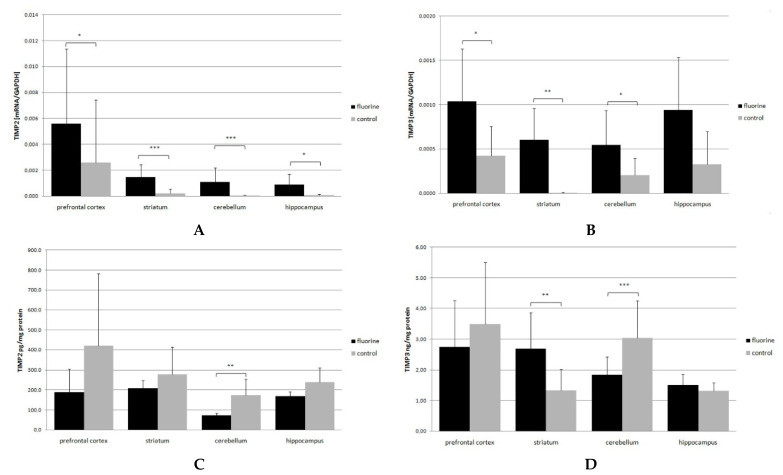
TIMP2 and TIMP3 mRNA (**A**,**B**) and protein (**C**,**D**) expression in the prefrontal cortex, striatum, cerebellum and hippocampus. Black bars represent means ± SD of protein (**A**,**B**)/mRNA (**C**,**D**) level in F-exposed group, while gray bars represent controls. * *p* ≤ 0.05, ** *p* ≤ 0.005, *** *p* ≤ 0.001 (Mann–Whitney U test), *n* = 6.

In the prefrontal cortex, TIMP2 mRNA expression in the F-exposed group was stronger by 116% (*p* = 0.034), in the striatum it was more than 6 times higher (*p* = 0.0002), in the cerebellum it was more than 30 times higher (*p* = 0.00001), and in the hippocampus it was more than 13 times higher (*p* = 0.016) than in the control (Figure 4C). Similar results were obtained for the TIMP3 mRNA level in the prefrontal cortex (more than 300%, *p* = 0.034), in the striatum (*p* = 0.004) and in the cerebellum (162%, *p* = 0.034) (Figure 4D).

The level of TIMP2 protein was lower in all the brain structures of interest in the F-exposed group compared to the control, but the cerebellum was the only structure with a statistically significant difference (*p* = 0.006), amounting to 58.5% (prefrontal cortex—55%, striatum—25%, hippocampus—29.5%) (Figure 4A).

At the same time, a significantly lower level of TIMP3 protein, down by 39%, was detected in the cerebellum of F-exposed rats (*p* = 0.0005). With respect to the striatum, a significantly higher level of TIMP3 was demonstrated, by as much as 101% (*p* = 0.0026) (Figure 4B). The differences in the TIMP2 gene expression were statistically significant across all the structures.

**Figure 5:**
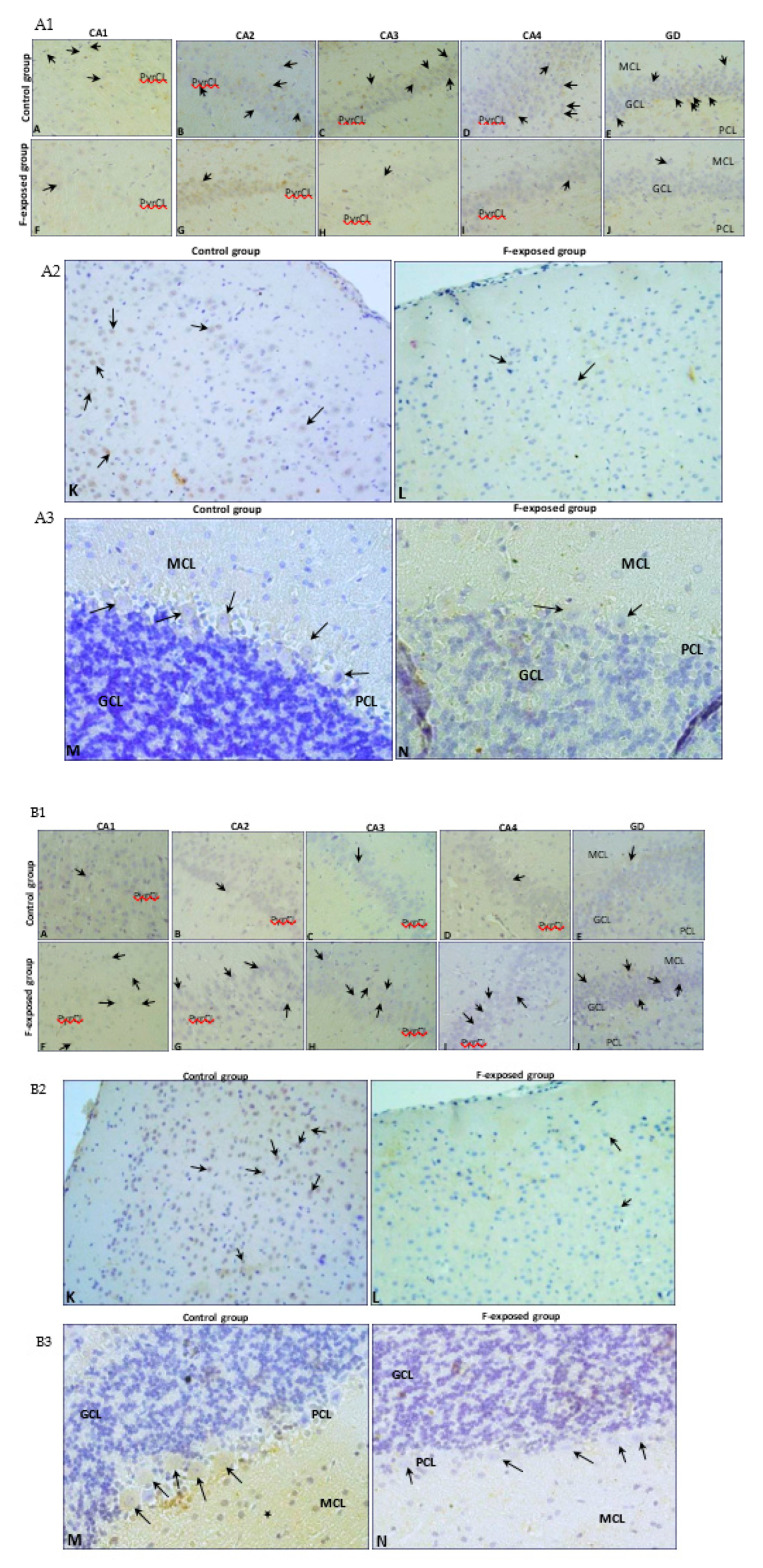
Immunolocalization of TIMP2 (**A1**–**A3**) and TIMP9 (**B1**–**B3**) in analyzed brain structures (hippocampus, objective magnification ×40 (**A1**,**B1**); prefrontal cortex, objective magnification ×20 (**A2**,**B2**); cerebellum, objective magnification ×20 (**A3**,**B3**)). Hippocampus (CA1–CA4): area of cornu ammonis (CA); gyrus dentate (GD); pyramidal cell layer (PyrCL); granular cell layer (GCL); polymorphic cell layer (PCL); molecular cell layer (MCL).

**Figure 6:**
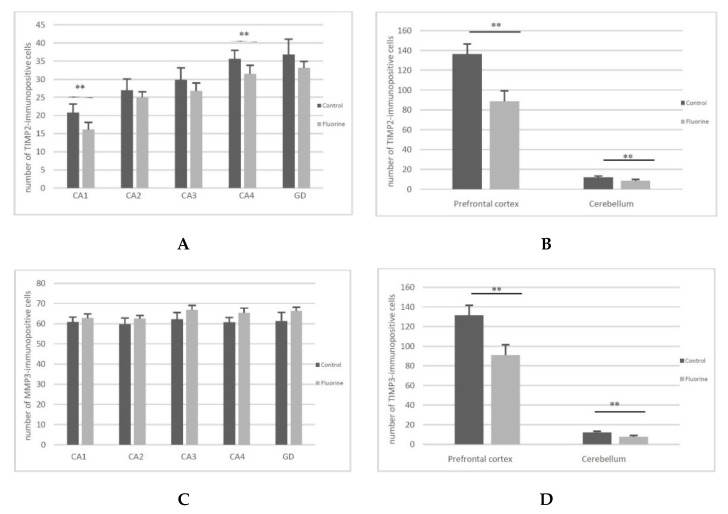
The number of TIMP2-immunopositive cells in the hippocampus (**A**) and in the prefrontal cortex and cerebellum (**B**) and the number of TIMP3-immunopositive cells in the hippocampus (**C**) and in the prefrontal cortex and cerebellum (**D**). ** *p* ≤ 0.005 (Mann–Whitney U test), *n* = 6.

All hippocampal regions of both groups showed a positive reaction confirming the presence of TIMP2 and TIMP3. However, no significant differences in the expression were observed between the control (Figure 5, TIMP2 A-E, black arrows; TIMP3 A–E, black arrows) and the study groups (Figure 5, TIMP2 F–J, black arrows; TIMP3 F–J, black arrows). We observed a statistically significant decreased number of TIMP2-immunopositive cells in CA1 (*p* = 0.009) and CA4 (*p* = 0.015) regions of hippocampus (Figure 6A). The number of TIMP3-immunopositive cells was increased in all hippocampal regions, but differences were not statistically significant (Figure 6C).

In the prefrontal cortex, a stronger TIMP2 and TIMP3 expression was observed in the perinuclear cytoplasm of neurons in the control group (Figure 5, TIMP2 K, black arrows; TIMP3 K, black arrows) compared to the study group (Figure 5, TIMP2 L, black arrow; TIMP3 L, black arrow). The number of TIMP2- and TIMP3-immunopositive cells in the prefrontal cortex was significantly decreased (*p* = 0.002; *p* = 0.002) (Figure 6B,D).

In addition, stronger TIMP2 reaction intensity in the perinuclear cytoplasm of neurons in the control group (Figure 5, TIMP2 M, black arrows) compared to the study group (Figure 5, TIMP2 N, black arrow) was detected in the cerebellum. More intensive expression of TIMP3 was shown in the cytoplasm of Purkinje cells in the cerebellum (Figure 5, TIMP3 M, black arrows), as well as in the cytoplasm of neuroglial cells in the control group compared to the study group (Figure 5, TIMP3 N, black arrow). The number of TIMP2- and TIMP3-immunopositive cells in the cerebellum was significantly decreased (*p* = 0.002; *p* = 0.002) (Figure 6B,D).

## 4. Discussion

### 4.1. Fluoride Accumulates Selectively in Different Parts of the Brain

The test animals used in our study were exposed to 50 mg/L aqueous solution of sodium fluoride which they ingested in drinking water. The dosage of NaF used in the present experiment was calculated based on research findings by other scholars, so as to achieve a similar F concentration in serum as that found in people with environmental F exposure [45,46]. Such a research model is often used in F toxicology studies, and 50 mg/L NaF is regarded as a relatively low dose [47,48,49]. The control group was given ordinary tap water to drink, in which the level of F ions, in line with the provisions of Polish law concerning the acceptable mineral concentrations in drinking water, did not exceed 1.5 mg/L. The dosage used in this experiment protocol was low enough not to cause significant changes in serum F levels determined in the rats from the study group compared to the control. On the other hand, the level of F ions in drinking water combined with the long-term exposure of the rats to the mineral, starting in the prenatal period and continuing throughout their lifetime, until reaching full sexual maturity (at PSD 90), resulted in a significant accumulation of F in their brains.

As we showed before in our previous research [50], the experimental model used in the present study caused the F level in serum to be similar in both groups (F-exposed group 0.19 mg/L vs. control 0.18 mg/L). All the studied brain structures were found to contain a higher F level in the F-exposed group (range 80–200 mg/kg) compared to the control (range 70–150 mg/kg). A study using a similar experimental model has shown that 50mg/L of NaF causes fluoride accumulation in the hippocampus [51], while in another study researchers got a significantly higher level of plasma F [52] using the same dosage of F. The reason why the results are different may be the different time points of tissue collection (PSD 90 in our study vs. PSD 21) and the design of control. Since the cited studies used deionized water as a control, while having in mind that metalloproteinases and all brain processes are sensitive to lack of basic anions and cations, we decided to use tap water as the control and base water for NaF concentration for the experimental group for better reflection of the processes in a normal environment. 

### 4.2. Fluoride Changes Gene and Protein Expression of Metalloproteinases

This is a pioneering study, the first to address the question of in vivo effects of low-dose F on the expression of metalloproteinases MMP2 and MMP9 in selected brain structures. Our findings demonstrate that F exposure alters the gene and protein expression of MMP2 and MMP9. Previous reports on the influence of sodium F on inhibiting metalloproteinase activity dealt with human saliva. An in vitro study with purified enzymes showed that F at 200 ppm can completely inhibit the activity of MMP9 and MMP2, with partially reversible inhibition of the enzymes at 250–1500 ppm and irreversible inhibition at 5000 ppm F [53]. In an animal model study, it was observed that F intake changes the expression of MMP2 and TIMP-1 in the extracellular bone matrix, affecting bone remodeling processes [54].

Metalloproteinases play an important role in regulating neuroplasticity processes. In normal physiology, MMP/TIMP-mediated proteolysis of ECM components accompany the reorganization and formation of new synaptic connections in the development of cognitive function [55]. People exposed to F at an early stage of development manifest a reduced intelligence quotient and cognitive impairment [31,32,33,34,35,36,37,56,57,58,59]. Metalloproteinases affect the maturation of neuronal spines, e.g., MMP9 has been demonstrated to contribute to the structural and functional reorganization of excitatory synapses located on dendritic spines [60]. MMP9 deletion causes early postnatal development disorders, increasing the number of CA1 pyramidal neurons and at the same time decreasing dendritic length and complexity; individual CA1 neurons in MMP9^−/−^ mice have enhanced input resistance and a significant increase in the frequency, but not amplitude, of miniature excitatory postsynaptic currents (mEPSCs) [61]. In the cited paper, parallel changes were observed in mature individuals in the neurons of the primary visual cortex, suggesting that the enzyme has a role to play not only in the formation and maturation of neuronal circuits in early life but also in adulthood. This is confirmed by the findings from a study on rats in which learning impairment was observed as a result of the administration of MMP9 inhibitors [62]. In a study investigating the plasticity of the somatosensory cortex, it was demonstrated that LTP induced by the spike timing-dependent plasticity (STDP) paradigm is strongly correlated with associative learning and critically depends on the activity of MMP9 [63]. It was also reported that the expression of MMP2 and MMP9 (mRNA and protein concentration) differs in people with recurrent depressive disorders and impaired cognitive functions compared to healthy controls [64]. MMP2 KO mice were found to exhibit increased anxiety and differences in motor activities in behavioral tests, suggesting that MMP2 plays an important role in cognitive and motor behaviors [65]. The changes in the levels of MMP2 and MMP9 (mRNA and protein) observed by us were correlated with the accumulation of F in the brain structures of key importance for the development of cognitive processes. It may therefore be suspected that the observed changes in the MMP/TIMP balance are involved in the cognitive impairment accompanying F intoxication.

MMPs may also interact with other molecules at the site of nerve tissue damage. High concentrations of F promote neuronal apoptosis and necrosis [1,66]. At the site of brain damage, the synthesis of nitric oxide (NO) is upregulated, which may contribute to further tissue degradation. NO activates MMP9 through S-nitrosylation, leading to neurotoxic effects [67]. Nerve damage, as a result of a stroke or mechanical injury to the spinal cord, triggers increased infiltration of neutrophils to the CNS [68,69]. While elevated concentrations of the metalloproteinases of interest result from their enhanced synthesis within the CNS, they may also be released by immune cells, induced by the systemic effects of fluoride toxicity. Neutrophils recruited to the site of tissue injury release large amounts of MMP9 from their granules [70], and that is why we can observe higher levels of their proteins at the site of injury without increased transcription in nerve tissue [71].

The MMP/TIMP alterations observed in the present study may suggest a disruption of the blood-brain barrier (BBB). This is because MMPs play a key role in maintaining BBB integrity, and their increased activity leads to BBB damage [72]. Therefore, bearing in mind that fluorides can pass through the BBB, initiating an inflammatory state in the brain, which involves MMP activation mediated by inflammatory chemokines and cytokines, it may be concluded that the process takes the form of a positive feedback loop. The consequences of such a state include a weakened BBB, making it even easier for the mineral to pass into the brain. The constant influx of F through the compromised BBB leads to chronic inflammation and enhanced neurotoxic effects.

In our study, we found a significantly lower level of TIMP2 in the F-exposed group compared to the control. TIMP2 is a selective inhibitor of MMP2 and reduces the loss of BBB integrity resulting from the proteolytic activity of that metalloproteinase [73]. Thus, it may be concluded that in the experimental group the BBB was compromised.

F-induced disruption of the BBB, increased ROS synthesis and development of an inflammatory state are the direct causes of brain damage. Both MMP2 and MMP9 participate in tissue regeneration following injury, due to their ability to degrade ECM components. In a study investigating the regeneration of damaged nerve fibers, increased levels of MMP9 were observed following brain injury, which was correlated with the development of inflammatory response, neuron degradation and disruption of the blood-brain barrier [24]. In a subsequent study focusing on olfactory nerve injury, it was demonstrated that the increase of MMP9 as a result of nerve transection is followed by a rise in MMP2 during recovery and neuronal regeneration [25]. MMP2 levels change independently of MMP9, probably due to the significant role of that metalloproteinase in axonal regeneration processes [25]. The findings observed in the above experiment have been confirmed in studies carried out by other research teams, and they seem to confirm the role of MMP2 in regulating neurite growth [74,75]. In a study on kainate-induced neuronal damage, increased activity of MMP9 was observed in hippocampal neurons with simultaneous broad-spectrum protective effects of TIMPs [76]. In a similar experimental model, increased activity of MMP9 in astrocytes was associated with a decrease in laminin immunoreactivity in the ganglion cell layer and led to increased apoptosis of retinal ganglion cells *(RGCs)* [77].

Taking into consideration the relationships described above, it may be suspected that increased synthesis of MMP2 in F-exposed brains may be related to the regeneration processes in response to CNS damage initiated by the mineral. The structure-specific changes in MMP9 levels in the experimental group (up in the striatum, down in the cerebellum) may be due to the chronic nature of F toxicity. Increased levels of the enzyme are typical of the initial stages of nerve tissue regeneration. The ambiguity of our findings suggests that the processes of tissue damage and regeneration in the brain are simultaneous and intertwined.

### 4.3. Fluoride Upregulates Gene Expression of Metalloproteinase Inhibitors in the Brain

In our study, we observed that F exposure significantly affected the quantities of synthesized TIMP2 and TIMP3, as well as the expression of the relevant encoding genes. One of the reasons for the observed changes in inhibitor synthesis may be their different form in the brain. TIMP2 as a soluble protein may be degraded more rapidly, and the changes in its level will be easier to detect. TIMP3 breaks down at a slower rate, and its actual amounts may be more difficult to monitor over time because of its close association with the ECM [78,79]. What is more, F-induced increases of mRNA quantities for TIMP2 and TIMP3 appear to be a response to the decrease in TIMP2 proteins and partly TIMP3 (prefrontal cortex and cerebellum) compared to the control.

TIMPs act as MMP inhibitors by binding non-covalently to the catalytic domain of the enzyme. Reduced amounts of TIMPs may therefore result from the increased catalytical activity of MMPs and, conversely, increased levels of the inhibitors may point to suppressed MMP activity. TIMPs also have metalloproteinase-independent functions, playing a regulatory role in some cellular processes, such as cell proliferation and apoptosis, and angiogenesis [28,29,30]. Thus, changes in mRNA and protein expression of TIMPs may have a significant impact on a range of important processes in the brain.

TIMP2 is the specific inhibitor of MMP2 [28]. The markedly decreased level of TIMP2 observed in the brains of F-exposed rats, with a simultaneous increase of MMP2 suggests that the inhibition of the metalloproteinase was downregulated. It may therefore be concluded that F not only significantly contributes to increased MMP2 levels in the brain but may also stimulate its activity by suppressing the synthesis of its inhibitor TIMP2. TIMP3, on the other hand, is a broad-spectrum inhibitor of MMPs [28] and probably the main regulator of MMP activity [80]. Changes in the expression of the studied inhibitors may result in disruptions of special ECM proteolysis, which may in turn hinder the formation of new synaptic connections [81,82]. This view is also supported by the study suggesting that MMP inhibition inhibits neuronal growth cone activity [83]. What is more, TIMP3 plays a very important role in neurogenesis: it was observed that in the rostral migratory stream to the olfactory bulb TIMP3 is expressed in a pattern similar to one of the key proteoglycans forming perineuronal nets (PNNs)—brevican [84]. It was also demonstrated that apart from being a metalloproteinase inhibitor, TIMP3 suppresses other enzymes (including ADAMTS4 and ADAMTS3) which alongside MMPs are responsible for degrading another essential proteoglycan in PNNs—aggrecan [85,86,87]. Excessive suppression of proteoglycan degradation in PNNs deters new synapse formation [88]. Quantitative alterations in mRNA and protein expression of TIMPs seem to confirm that one of the key mechanisms behind the development of cognitive disorders, induced by F accumulation in brain tissue, is the abnormal activity of the MMP/TIMP enzymatic system.

In vitro studies showed that cerebral endothelium is crucial for maintaining the right balance between MMPs and TIMPs in the central nervous system which may be disturbed as a result of changes in glucocorticoid-regulated pathways [89,90]. Hydrocortisone was implicated in the upregulated synthesis of TIMP3 in cerebral ECM, which may be directly related to maintaining BBB integrity [91]. By inducing oxidative stress, F may increase cortisol secretion, via an ATP-dependent ion transport mechanism [92]. It may therefore be presumed that elevated levels of TIMP3 protein in the striatum and hippocampus are related to the protective effects of cortisol on the BBB in response to the stress induced by F intoxication.

TIMPs are also implicated in apoptosis. So far, there are no conclusive research findings as to the exact role of TIMP2 in that process [93,94]. TIMP3, on the other hand, is involved in inducing apoptosis by modulating the FAS death receptor [95], a mechanism that has been confirmed for a variety of cell types [96,97,98]. F in higher concentrations was demonstrated to trigger apoptosis in brain cells [99]. Our findings suggest that this correlation may be linked to the amounts and activity of TIMP2 and TIMP3.

In summary, F exposure during pre- and postnatal brain development may disrupt the MMP/TIMP enzymatic system in the hippocampus, prefrontal cortex, striatum and cerebellum. These disruptions may then impair neuroplasticity and cognitive function development, as observed by other scholars in humans and experimental animals exposed to fluoride intoxication. Moreover, the changes in mRNA and protein expression of MMP2, -3 and TIMP2, -3 observed in this study may point to an ongoing persistent inflammatory process in the brain and intertwined processes of brain tissue degeneration and regeneration.

## 5. Conclusions

In the present study, we found that long-term exposure to F causes imbalance in MMP2, MMP9, TIMP2 and TIMP3 protein and gene expression in such brain structures as the cerebellum, striatum, prefrontal cortex and hippocampus. Observed changes may be a key player in the molecular mechanism involved in cognitive deficits observed during fluoride intoxication.

## Data Availability

Data is contained within the article.
